# Impact of GLP-1 Receptor Agonists on Whole-Gut Gastrointestinal Motility Using Wireless Motility Capsule: A Descriptive Single-Center Case Series

**DOI:** 10.14309/crj.0000000000001789

**Published:** 2025-08-04

**Authors:** Michael Cymbal, Zehra Naseem, Din Hoxha, Samita Garg

**Affiliations:** 1 Department of Internal Medicine, Cleveland Clinic Foundation, Cleveland, Ohio; 2 Department of Gastroenterology, Hepatology, and Nutrition, Digestive Disease Institute, Cleveland Clinic Foundation, Cleveland, Ohio

**Keywords:** GLP-1 receptor agonists, gastrointestinal motility, wireless motility capsule, SmartPill

## Abstract

This retrospective case series investigates the impact of GLP-1 receptor agonists (GLP-1RAs) on whole-gut gastrointestinal motility using wireless motility capsule testing. We analyzed 10 patients on GLP-1RAs who underwent wireless motility capsule testing for constipation or gastroparesis to assess gastric, small bowel, and colonic transit times. Delayed gastric emptying time was observed in 80% of patients, whereas delayed whole-gut transit time was noted in 44% of patients. The 3 patients on semaglutide (1 mg) had the longest gastric emptying time, delayed whole-gut transit time, and were the only patients with delayed small bowel transit time. Although limited by retrospective observation, our findings are consistent with the effects of GLP-1RAs on gastric motility delay and suggest an impact on whole-gut motility. With the expanding use of GLP-1RAs, these findings underscore the need for prospective studies using emerging motility technologies to further characterize the motility effects both during treatment and after cessation, thereby guiding evidence-based clinical decision-making.

## INTRODUCTION

Glucagon-like peptide-1 receptor agonists (GLP-1RAs), specifically exenatide, were initially approved by the Food and Drug Administration (FDA) in 2005 for type 2 diabetes mellitus (T2D).^1^ Over the past 2 decades, GLP-1RA use has grown exponentially for both T2D and obesity, and numerous studies have shown improvement in cardiovascular and kidney outcomes.^2–8^

GLP-1RA is primarily secreted from enteroendocrine L cells in the distal ileum and colon, with receptors throughout the gastrointestinal (GI) tract.^2^ Although secretion and receptor location are primarily in the GI tract, effects from GLP1-RAs are both peripheral and central. Centrally, effects work to regulate appetite, reduce hunger, and increase satiety.^2^ Peripherally, GLP-1RAs enhance glucose-dependent insulin release from beta-cells, inhibit paradoxical glucagon release from alpha cells, and delay gastric emptying.^2^

The effects on GI motility occur through the inhibition of myenteric neurons and a reduction in small bowel migrating motor complexes activity.^9^ Although there is an understanding of the mechanisms of delayed GI motility, the specific effects on gastric, small bowel, and colonic motility are not well-defined. One validated method for evaluating GI motility is the wireless motility capsule (WMC), a noninvasive ambulatory study in which a small, nondigestible capsule is swallowed and transmits data regarding luminal pressures, temperatures, and pH values to a receiver worn around the patient's waist.^10,11^ WMC allows for the assessment of key motility measures, including gastric emptying time (GET), small bowel transit time (SBTT), colon transit time (CTT), and whole-gut transit time (WGTT).^10,11^ Delayed motility is defined as GET ≥ 4-5 hours and severe delay >12 hours, SBTT ≥ 6 hours, CTT ≥ 59 hours, and delayed WGTT ≥ 73 hours.^10–12^

Motility abnormalities have been demonstrated in patients with long-standing or poorly controlled diabetes.^13^ This study aimed to evaluate the effect of GLP-1RAs on gastric, small bowel, and colonic motility using WMC.

## METHODS

We performed a single-center, retrospective review of patients 18 years or older who were receiving GLP-1RAs before and during the WMC study completion, from January 2018 to December 2023, at the Cleveland Clinic. Patients were identified through procedural codes and medication prescription history. Three reviewers performed an extensive chart review to exclude patients who stopped GLP-1RAs before the testing date, had prior bowel surgery or were taking scheduled laxatives or motility medications. We evaluated demographics, GLP-1RAs type, dosages, duration of therapy, testing indication, WMC parameters, and previous motility testing. Potential confounding factors were also assessed, including additional GI motility medications, hypothyroidism, neurological conditions, and duration and control of T2D.

## RESULTS

Initially, 74 patients were identified through procedural codes and medication prescription histories, of which 10 patients, 7 male and 3 female, met the criteria for further evaluation based on exclusion and inclusion criteria. The mean age of the patients was 54.0 years, with a standard deviation (SD) of ±11.3 years, and an average body mass index of 33.8 ± 7.1 (Table 1). Semaglutide was the most prevalent GLP-1RA (n = 5), followed by dulaglutide (n = 4). The most common indication (n = 8) for the WMC study was for gastroparesis with a previous gastric emptying study, and 2 patients had chronic constipation.

**Table 1.** Demographics of patients from the study

#	Age	Gender	Race	BMI at study	T2D (Y/N) (HbA1c at study)	T2D Diagnosis Year (years before study)	Hypothyroid (TSH)	Opioid as needed Y/N
1	50	Male	White	30.3	Y-(6.2)	2017 (5)	N/A	N
2	49	Female	Black	28.6	Y-(6.2)	2018 (4)	N/A	N
3	50	Female	Black	26.7	N- (5.3)	N/A	N/A	N
4	46	Female	White	44.3	Y-(6.7)	2017 (4)	N/A	N
5	66	Female	White	29.5	Y-(6.7)	1998 (23)	N/A	N
6	75	Male	White	28.5	Y-(5.8)	2007 (16)	Y- (2.2)	N
7	44	Female	White	35.0	Y-(6.4)	2021 (1)	N/A	Y
8	40	Female	Multiracial	47.7	Y-(6.8)	2009 (13)	N/A	Y
9	54	Female	White	31.6	Y-(6.5)	2022 (1)	N/A	N
10	66	Female	White	36.2	Y- (6.3)	2007 (15)	N/A	N

BMI, body mass index; T2D, type 2 diabetes mellitus.

All patients who met criteria for further analysis were not on laxatives, and 2 were prescribed low-dose as-needed opiates that were held 1 week before the test date. Overall, 90% of the patients had T2D, 5 of whom were diagnosed < 5 years before the test date, and 4 were diagnosed > 10 years before. T2D was well-controlled at the time of the study, with a mean HbA1c of 6.4 ± 0.32%. None of the patients had the Parkinson disease, and 1 patient had well-controlled hypothyroidism (TSH 2.2 mIU/L). Nine patients completed previous gastric emptying scintigraphy (GES) testing, all of whom were taking GLP-1RAs at the time of GES.

The mean and median values for prescription durations before the study were as follows: the duration of GLP-1RA before the study was 782.6 ± 546.5 days (mean ± SD) and 742 days (median). Transit times in hours: minutes, GET 19:16 ± 13:55 (mean ± SD) and 21:01 (median); SBTT 6:28 ± 6:12 (mean ± SD) and 4:25 (median); CTT 45:11 ± 36:45 (mean ± SD) and 46:13 (median); and WGTT 70:56 ± 44:51 (mean ± SD) and 71:58 (median) (Table 2). Mean transit time for semaglutide (n = 4): GET 26:16, SBTT: 9:40, CTT 55:34, WGTT 91:45; dulaglutide (n = 4) GET 16:07, SBTT 3:48, CTT 43:41, WGTT 63:38.

**Table 2.** Details of GLP-1 agonists, duration, and doses in relation to SmartPill motility study transit times

#	Indication	GLP-1 RA	Dose (mg) at study	Duration of GLP1-RA before study (days)	GET (hr: min) ≥ 4-5 hours suggests delayed gastric emptying	SBTT (hr: min) ≥ 6 hours suggests delayed transit	CTT (hr: min) ≥ 59 hours suggests delayed colonic transit	WGTT (hr: min) ≥ 73 hours suggests delayed transit	Prior GES, % retained at 4 hours (GLP1-RA, Y/N)
1	Gastroparesis	Dulaglutide	1.5	515	17:38 - SDT	4:36	21:13	43:27	12 (Y)
2	Gastroparesis	Dulaglutide	1.5	1,376	4:34- D	3:01	97:14- D	104:49-D	45 (Y)
3	Gastroparesis	Semaglutide	0.25	44	Pill retention				N/A
4	Constipation	Exenatide	2.0	904	3:44	4:25	9:47	17:57	18 (Y)
5	Gastroparesis	Dulaglutide	1.5	742	21:01- SDT	3:43	9:32	34:17	39 (Y)
6	Gastroparesis	Semaglutide	1.0	1811	25:01- SDT	22:48 -D	46:13	94:02 -D	79 (Y)
7	Gastroparesis	Semaglutide	0.25	111	4:07- D	3:41	9:25	17:14	44 (Y)
8	Gastroparesis	Semaglutide	1.0	248	25:59- SDT	6:01- D	103:26- D	136:27-D	15 (Y)
9	Constipation	Semaglutide	1.0	415	49:56- SDT	6:09- D	63:10- D	119:16-D	90 (Y)
10	Gastroparesis	Dulaglutide	4.5	922	21:23- SDT	3:50	46:44	71:58	76 (Y)

CTT, colon transit time; D, delayed; GET, gastric emptying time; SBTT, small bowel transit time; SDT, severe delayed time; WGTT, whole-gut transit time.

## DISCUSSION

In this retrospective cohort, we found that 90% of patients using GLP-1RAs exhibited significant delays in GI motility on WMC testing. The most prevalent abnormality was delayed gastric emptying, affecting 80% of patients, followed by small bowel transit and colon transit delay in 33% of patients. Forty-four percent of patients demonstrated delayed WGTT. Importantly, delay in motility was observed even in patients with well-controlled T2D, suggesting the GLP-1RA-related dysmotility may be independent of glycemic control.

These results from WMC are consistent with delays in gastric scintigraphy which was abnormal in 8 of the 9 patients who received GES testing on GLP-1RAs. This aligns with data from the SUSTAIN 7 trial, which found semaglutide and dulaglutide were associated with high rates of GI adverse events (33% and 48%, respectively).^14^ Our analysis showed a stronger association of GET and WGTT delays in patients receiving semaglutide compared with dulaglutide at comparable doses.

In addition, the dose and duration of GLP-1RA also appear to be relevant. We found that 50% of the patients had recently increased their dose of GLP-1RA before the study, and 70% had been on GLP-1RAs for at least 1 year. All 7 patients receiving dulaglutide ≥ 1.5 mg (4) or semaglutide 1 mg (3) had delayed GET or WGTT and accounted for all 6 patients who had severe delayed GET, defined as greater than 12 hours.^11^ The 3 patients who received the increased dose of semaglutide (1 mg) all exhibited delayed WGTT and had the 3 longest GET measurements. They were the only patients who exhibited delayed SBTT, although in 2 patients, the delay was minimal. SBTT was less frequently affected overall compared with gastric and colon transit times. Interestingly, the fastest WGTT was observed in a patient on the lowest semaglutide dose (0.25 mg) and shortest treatment duration (111 days). The only patient receiving exenatide showed no identifiable motility delay. No patients in this study were treated with tirzepatide, which warrants further research in the future.

Despite the discontinuation of Smartpill WMC manufacturing in June 2023, our tertiary center maintained the largest Smartpill database in the United States, with continued use through the end of 2024. WMC offered comprehensive whole-gut motility data, which remain unmatched by current testing modalities (GES for gastric emptying, video capsule endoscopy for SBTT, and sitz marker studies for colonic transit), each with inherent limitations. This underscores the value of whole-gut assessment tools.

Emerging technologies such as MotiliCap, a next-generation whole-gut transit capsule, and its associated software, MotiliScan, recently received FDA clearance (May 27, 2025) and now offer a new avenue to evaluate GI transit.^15^ Other innovations, such as the Atmo Gas Capsule, pending FDA approval, may further advance the field.^16^ The clinical relevance of comprehensive motility assessment is increasingly important, given the growing prevalence of patients with symptomatic alterations in motility associated with GLP-1RAs and the rapidly increasing rate of their prescription.^17^

Despite limitations inherent in its retrospective design, this study provides valuable data showing the changes in whole-gut motility on GLP-1RAs. These findings underscore the need for prospective studies with emerging technologies evaluating whole-gut motility in patients using newer GLP analogs, incorporating testing both during GLP treatment and after cessation. Such studies would help inform both patients and healthcare providers about the GI risks associated with GLP-1RAs to guide symptom expectations, management, and informed clinical decision-making.

## DISCLOSURES

Author contributions: M. Cymbal: drafting the article; Z. Naseem, D. Hoxha: critical revision of the manuscript; S. Garg: final approval of the manuscript and is the article guarantor.

Financial disclosure: None to report.

Informed consent was obtained for this case report.

## ABBREVIATIONS:

BMI: body mass index
CTT: colon transit time
D: delyaed
FDA: Food and Drug Administration
GES: gastric emptying scintigraphy
GET: gastric emptying time
GI: gastrointestinal
GLP-1RAs: Glucagon-like peptide-1 receptor agonists
HbA1c: hemoglobin A1c
SBTT: small bowel transit time
SD: standard deviation
SDT: severe delayed time
T2DM: type 2 diabetes mellitus
TSH: thyroid-stimulating hormone
WGTT: whole-gut transit time
WMC: wireless motility capsule
